# Angiopoietin‐like protein 3 complete and partial deficiency markedly accelerates apolipoprotein B48 and B100 metabolism in triglyceride‐rich lipoproteins in humans

**DOI:** 10.1111/joim.70124

**Published:** 2026-06-11

**Authors:** Marcello Arca, Elias Björnson, Laura D'Erasmo, Alessia Di Costanzo, Simone Bini, Ilenia Minicocci, Daniele Tramontano, Stella Covino, Giuseppe Ciarlo, Manuela Lombardi, Sanni Söderlund, Niina Matikainen, Linda Andersson, Martin Adiels, Marja‐Riitta Taskinen, Chris J. Packard, Jan Borén

**Affiliations:** ^1^ Department of Translational and Precision Medicine Sapienza University of Rome Rome Italy; ^2^ Department of Molecular and Clinical Medicine, Institute of Medicine University of Gothenburg Gothenburg Sweden; ^3^ San Giovanni di Dio, Fondi Hospital, ASL Latina Fondi Italy; ^4^ Research Programs Unit, Clinical and Molecular Medicine University of Helsinki Helsinki Finland; ^5^ Endocrinology, Abdominal Center Helsinki University Hospital Helsinki Finland; ^6^ Institute of Cardiovascular and Medical Sciences University of Glasgow Glasgow UK

**Keywords:** ANGPTL3 (angiopoietin‐like protein 3), apoB, kinetics, loss‐of‐function variants, triglyceride‐rich lipoproteins (TRL)

## Abstract

**Background:**

Angiopoietin‐like protein 3 (ANGPTL3) is a key circulating regulator of triglyceride metabolism and a promising pharmacological target. The physiological consequences of profound ANGPTL3 deficiency can be explored in individuals with inherited loss‐of‐function (LOF) variants, who show reduced lifetime risk of atherosclerotic cardiovascular disease.

**Methods:**

ApoB48, apoB100 and TG metabolism were investigated in chylomicrons, VLDL1, VLDL2, IDL and LDL in 3 *ANGPTL3* LOF homozygotes (undetectable plasma ANGPTL3), 4 LOF heterozygotes (ANGPTL3 45.0 ± 7.4 ng/mL) and 10 matched controls (ANGPTL3 110.5 ± 27.6 ng/mL). Studies were performed under post‐prandial conditions to comprehensively characterize TG transport and apoB‐containing lipoprotein kinetics.

**Results:**

Chylomicron and very‐low‐density lipoproteins (VLDL) production rates were similar in ANGPTL3‐deficient subjects and controls. The defining abnormality in LOF homozygotes was the extremely rapid lipolysis of chylomicrons and VLDL, with circulating residence times of minutes rather than hours and accelerated conversion of VLDL to IDL and LDL. LDL particles in LOF homozygotes were TG‐enriched, cholesterol‐depleted, metabolically heterogeneous and cleared more rapidly than in controls or LOF heterozygotes. LOF heterozygotes showed a less pronounced increase in chylomicron and VLDL lipolysis, with normal IDL and LDL kinetics.

**Conclusion:**

Complete loss of ANGPTL3 results in a rapid acceleration of the triglyceride‐rich lipoprotein lipolysis pathway and promotes the formation of metabolically and compositionally abnormal LDL with accelerated clearance. These findings provide mechanistic insight into ANGPTL3 deficiency and are directly relevant for the development and safety assessment of ANGPTL3‐targeted therapies.

## Graphical abstract

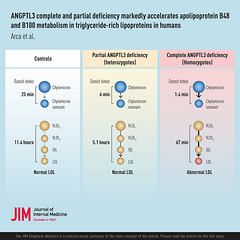

**Abbreviations**
ANGPTL3: angiopoietin‐like protein 3
apoB: apolipoprotein
CETP: cholesteryl ester transfer protein
EL: endothelial lipase
FCR: fractional clearance rate
FHBL2: familial combined hypolipidemia
HDL‐C: high‐density lipoprotein cholesterol
IDL: intermediate‐density lipoprotein
LDL‐C: low‐density lipoprotein cholesterol
LOF: loss‐of‐function
LPL: lipoprotein lipase
TRL: triglyceride‐rich lipoproteins
VLDL: very‐low‐density lipoprotein

## Introduction

Angiopoietin‐like protein 3 (ANGPTL3) has emerged as a key factor in the regulation of lipoprotein metabolism and triglyceride (TG) transport. It is synthesized in hepatocytes as a glycoprotein, secreted into the blood circulation and functions as an inhibitor of both lipoprotein lipase (LPL) and endothelial lipase (EL). It acts in a coordinated manner with ANGPTL4 and ANGPTL8 to determine the rate and site of LPL‐mediated hydrolysis of TG contained within triglyceride‐rich lipoproteins (TRL), chylomicrons and very‐low‐density lipoproteins (VLDL) [1, 2, 3]. Further, due to its inhibitory action on EL, an enzyme involved in the breakdown of high‐density lipoprotein (HDL) phospholipids, ANGPTL3 also regulates HDL levels in the circulation. Consistent with these functional roles, genetic and pharmacological inactivation of ANGPTL3 in mice is associated with de‐repression of LPL and EL activity, and significant reductions in plasma total TG and total cholesterol [4, 5, 6].

Recognition that TRL and their remnants are independent causal risk factors for atherosclerotic cardiovascular disease (ASCVD) has led to a search for targets that are amenable to pharmacological action. Several have been identified, principally apolipoprotein (apo) CIII and ANGPTLs 3, 4 and 8 [7, 8]. Agents that have the ability to alter profoundly the amount or activity of these proteins are in clinical development, but it is not yet clear which target will deliver the optimum combination of efficacy and safety, not only in terms of TRL lowering but also in ASCVD prevention. Previous investigations of the effects of ANGPTL3 inhibition have indicated that this intervention may lead to LDL lowering by a mechanism distinct from the LDL receptor pathway. Reeskamp et al. demonstrated in homozygous familial hypercholesterolemic (FH) subjects with no or very low LDL receptor activity that evinacumab could lower LDL substantially by increasing the clearance rate of the lipoprotein [9]. Animal model studies provided evidence that this may an EL‐mediated pathway so that in the absence of LDL receptors, the enzyme (whose activity is stimulated when ANGPTL3 is low) acts on LDL to promote its uptake by hepatocytes [10].

In humans, complete ANGPTL3 deficiency due to bi‐allelic loss‐of‐function (LOF) mutations in ANGPTL3 causes a form of familial combined hypolipidemia (FHBL2), a very rare lipid disorder characterized by reductions in LDL cholesterol (LDL‐C), HDL‐C and VLDL [11, 12]. FHBL2 is associated with increased LPL mass and activity in post‐heparin plasma [11, 12], and affected individuals have markedly decreased post‐prandial TG levels after a fat‐rich meal [13], lower plasma free fatty acids and improved insulin sensitivity [14, 15]. The lower concentrations of both LDL and HDL cholesterol distinguish inherited *ANGPTL3* LOF from the lipid phenotypes seen in ANGPTL4 and apoCIII deficiency states where LDL‐C appears to be normal and HDL‐C is elevated [16, 17]. Consistent with the favourable changes in plasma lipoprotein profile, it has been reported that carriers of *ANGPTL3* LOF mutations have a reduced atherosclerotic burden as well as a lower risk of ASCVD events [18, 19]. Based on these findings, ANGPTL3 has been identified as a promising pharmacological target to reduce ASCVD risk by modulating TRL metabolism [20, 21, 22]. However, the mechanisms underlying the reduction of apoB‐containing lipoproteins in FHBL2 are still a matter of debate. To date, investigations of lipoprotein kinetics in complete ANGPTL3 deficiency have revealed increased catabolism of LDL‐apoB, greatly increased catabolism of TRL‐apoB and evidence for a reduced production rate for VLDL‐apoB in the liver [23, 24].

The present study aimed to provide a more comprehensive evaluation of the changes in apolipoprotein B metabolism in subjects with both heterozygous and homozygous ANGPTL3 deficiency. A major objective was to elucidate in detail the impact of partial and complete lack of this regulatory protein on chylomicron, VLDL and LDL kinetics since pharmacological agents, such as RNA‐silencing drugs, do not fully eliminate ANGPTL3 but reduce it to levels between those seen in LOF heterozygous and homozygous individuals.

## Methods

### Subjects

The subjects of this study were nine men and eight women. ANGPTL3‐deficient participants were inhabitants of Campodimele, a village near Rome, Italy. Controls (normal ANGPTL3 variant) were recruited from Campodimele and from the Finnish population. Three subjects were homozygous and four were heterozygous for ANGPTL3 deficiency. All had the same S17X nonsense variant in exon 1 of the *ANGPTL3* gene, which has been characterised previously [12]. Heterozygotes were selected for study on the basis of a low plasma ANGPTL3 concentration to investigate the impact of low but not absent ANGPTL3 on apoB48 and apoB100 kinetics. Controls matched for age and body mass index (BMI) with the ANGPTL3‐deficient subjects had the normal variant of *ANGPTL3*. Controls from Finland were recruited from the THL Biobank (https://thl.fi/en/web/thl‐biobank/for‐researchers/sample‐collections). General inclusion criteria were age 20–75 years, BMI <35 kg/m^2^ and non‐smoking status. Exclusion criteria were history of any cardiovascular or severe disease, any condition affecting lipid levels, abnormalities in thyroid or kidney function or haematological abnormalities. None of the subjects used any medication or hormones known to influence lipid metabolism. The average plasma TG in the controls recruited to this study was very similar to that seen in the Campodimele normal variant inhabitants screened for possible inclusion (*n* = 28 with an average TG of 0.92 mmol/L, which is in line with an earlier report [25]).

### Metabolic study protocol

Metabolic studies were conducted at an outpatient clinic in the Hospital of Fondi (LT) and in the clinical research unit at the Helsinki University Central Hospital. Subjects were admitted at 8.00 AM after a 12 h fast, and the kinetics of apolipoprotein B‐48, B100, apo C‐III and apoE were determined using a tracer of deuterated leucine (5,5,5‐D3 Euriso‐Top, d3‐leucine) at a dose of 7 mg/kg body weight. The kinetics of triglyceride were determined using a tracer of deuterated glycerol (D1,1,2,3,3, D5 Euriso‐Top and d5‐glycerol) at a dose of 500 mg [26, 27]. Two hours after tracer administration, a standard fat‐rich meal (927 kcal) comprising bread, butter, cheese, ham, boiled egg, fresh red pepper, low‐fat (1%) milk, orange juice and tea or coffee (63 g carbohydrate, 69 g fat and 40 g protein) was consumed within 10 min. Blood samples were drawn before tracer injection and at frequent intervals thereafter until 10 h post‐administration, when a dinner was served. The subjects remained physically inactive, and only water was drunk (ad libitum) during the study. Further blood samples were obtained in the fasting state 24 h after tracer administration and then daily until 120 h after tracer administration.

### Lipoprotein isolation and biochemical analyses

Plasma from all blood samples acquired in Campodimele was packaged in Rome and transported on ice by air to Helsinki. The metabolic investigations were timed so sample collection, processing, shipment and delivery to the Helsinki lab occurred within 24–48 h. Thus, all lipoprotein isolations were conducted in the same laboratory using well‐established methods. Chylomicrons, VLDL1 (Sf 60–400) and VLDL2 (Sf 20–60) were isolated from blood samples by density gradient centrifugation [28], and IDL and LDL were prepared using sequential fixed density centrifugation [29]. Concentrations of triglycerides and cholesterol in total plasma and lipoprotein fractions were analysed using the Konelab 60i analyser (Thermo Fisher Scientific). Other laboratory tests were performed using standard methods. Plasma apoB48 and apoB100 were quantified by ELISA (Shibayagi). ApoB48 and apoB100 concentrations in lipoprotein fractions were measured by mass spectrometry as described previously [26]. Plasma apoC‐III was determined using an immunoturbidimetry‐based method (Kamiya Biochemical Company). ELISAs were used to measure serum apoE (STA‐367, Cell Biolabs) and ANGPTL3 (RD191092200R, BioVendor).

The terms ‘VLDL1‐apoB48’ and ‘VLDL2‐apoB48’ particles were adopted in earlier studies [26, 27, 29, 30] to describe lipoproteins that are isolated in the VLDL1 and VLDL2 density intervals that contain apoB48 (rather than apoB100). These lipoproteins are of intestinal origin and may represent particles generated by lipolysis of chylomicrons (remnants) but also VLDL‐sized lipoproteins secreted directly from the intestine [26, 27]. The terms are used as an operational definition as we cannot distinguish between these two origins (lipolysis and direct secretion) because the particles become labelled with the same tracer of deuterated leucine.

### Tracer enrichment in apolipoproteins and triglycerides, multicompartmental modelling and parameter estimation

The procedure for deriving kinetic rate constants using the non‐steady‐state compartmental model for apoB48/apoB100/triglyceride has been described in detail previously [26, 27, 29, 30]. The experimental protocol and modelling for apoC‐III have also been reported [29, 30]. Modelling and parameter estimation were performed using SAAMII [31]. Briefly, inputs to the model were the measured concentration of apoB48 in plasma, chylomicrons, VLDL1 and VLDL2; measured concentration of apoB100 in VLDL1, VLDL2, IDL and LDL; measured concentration of triglyceride in VLDL1 and VLDL2; tracer (deuterated leucine) enrichments in apoB48 in chylomicrons, VLDL1 and VLDL2; in apoB100 in VLDL1, VLDL2, IDL and LDL; and tracer enrichments (deuterated glycerol) in VLDL1 and VLDL2 triglyceride. Outputs from the model were production rates for apoB48, apoB100 and triglyceride in the relevant lipoprotein fractions, fractional clearance rates (FCRs) from each lipoprotein fraction and fractional transfer rates (FTRs) between precursor and product lipoproteins.

Non‐steady‐state compartmental models can accommodate changing tracee concentrations but require constraints to be introduced to permit calculation of rate constants. Our model permitted chylomicron TG and apoB48 concentrations to vary, as well as apoB48 and apoB100 concentrations in VLDL1 and VLDL2. Chylomicrons, as they appear in the circulation, compete with VLDL1 for available LPL, and so the VLDL1 to VLDL2 transfer (lipolysis) rate was permitted to vary as a function of time. VLDL1‐ and VLDL2‐apoB100 productions were constrained to a constant value over the period, and therefore the derived rates represent an average for each kinetic study (in theory, VLDL1 production could be influenced by fatty acid delivery to the liver during the post‐prandial period). Note that this approach does allow VLDL1‐ and VLDL2‐apoB production rates to vary between kinetic studies and so does not influence the ability to compare VLDL production rates between the groups of subjects.

### Statistical analyses

All statistical analyses were performed using R (version 4.0.2). *p* values for baseline characteristics in Table 1 were calculated using a *t*‐test for continuous variables and a chi‐square test for categorical variables (as per standard settings in package ‘tableone’). *p* values for kinetic parameters (Tables 2 and 3) were calculated using the Mann–Whitney *U* test using the ‘wilcox.test’ function in R. As we investigated small subject groups, the ability to conduct formal statistical comparisons was limited. However, for key variables we cite *p* values for comparisons between groups as a guide to the degree of difference observed and note where the findings are in line with differences described in larger cohorts of similarly affected subjects. All analyses used *n* = 10 for the control subjects except apoB48 kinetic analyses, where a technical error resulted in incomplete data for one participant.

**Table 1:** Subject characteristics and plasma lipid profile.

	ANGPTL3 def. homozygotes (*n* = 3)	ANGPTL3 def. heterozygotes (*n* = 4)	CONTROLS (*n* = 10)	*p* value
Sex (male/female)	1/2	2/2	6/4	
Age (years)	59.7 (11.9)	60.5 (8.9)	59.2 (9.0)	0.97
Height (m)	1.58 (0.053)	1.60 (0.084)	1.72 (1.000)	0.034
Weight (kg)	65.7 (8.1)	66.5 (11.5)	83.8 (12.6)	0.029
BMI (kg/m^2^)	26.4 (4.9)	25.6 (3.8)	28.2 (3.8)	0.50
Waist (cm)	97 (9)	92 (9.0)	102 (12)	0.33
Glucose (mmol/L)	5.8 (1.1)	4.7 (0.5)	5.2 (0.2)	0.037
Fasting insulin (IU)	6.5 (3.6)	11.0 (13.6)	5.4 (1.0)	0.38
**Plasma lipid profile**				
Plasma TG (mmol/L)	0.52 (0.12)	0.84 (0.36)	0.83 (0.20)	0.15
Plasma chol (mmol/L)	2.44 (0.33)	4.63 (0.48)	4.68 (0.44)	<0.001
LDL‐C (mmol/L)^a^	1.46 (0.32)	2.94 (0.50)	3.00 (0.25)	<0.001
HDL‐C (mmol/L)	0.68 (0.14)	1.4 (0.50)	1.58 (0.25)	0.002
ApoA1 (mg/dL)	79 (10.8)	134 (11.9)	141 (24.0)	0.002
Apo B (mg/dL)	55.6 (8.5)	77.7 (15.1)	81.8 (9.6)	0.009
ApoCIII (mg/dL)	1.32 (0.78)	7.5 (1.8)	8.7 (1.3)	<0.001
ANGPTL3 (ng/mL)	0.0 (0.0)	45.0 (7.4)	110.5 (27.6)	<0.001
**Basal lipoprotein lipid profile**				
Chylo TG (mmol/L)	0.002 (0.004)	0.058 (0.060)	0.038 (0.025)	0.15
VLDL1 TG (mmol/L)	0.009 (0.002)	0.183 (0.170)	0.302 (0.169)	0.037
VLDL2 TG (mmol/L)	0.012 (0.005)	0.128 (0.079)	0.195 (0.140)	0.092
IDL TG (mmol/L)	0.022 (0.018)	0.037 (0.020)	0.069 (0.096)	0.60
LDL TG (mmol/L)	0.293 (0.038)	0.176 (0.027)	0.179 (0.055)	0.007
Chylo chol (mmol/L)	0.001 (0.001)	0.018 (0.016)	0.005 (0.004)	0.031
VLDL1 chol (mmol/L)	0.005 (0.001)	0.078 (0.067)	0.072 (0.047)	0.116
VLDL2 chol (mmol/L)	0.010 (0.006)	0.117 (0.064)	0.235 (0.122)	0.012
IDL chol (mmol/L)	0.020 (0.019)	0.080 (0.054)	0.138 (0.194)	0.51
LDL chol (mmol/L)	1.43 (0.29)	2.44 (0.42)	2.56 (0.24)	<0.001
VLDL1 TG/chol ratio	1.52 (1.31)	2.23 (0.21)	4.28 (0.97)	<0.001
VLDL2 TG/chol ratio	1.09 (0.13)	0.96 (0.22)	1.08 (0.27)	0.72
IDL TG/chol ratio	2.44 (2.24)	0.55 (0.21)	0.56 (0.23)	0.015
LDL TG/chol ratio	0.21 (0.04)	0.08 (0.01)	0.07 (0.02)	<0.001
**ApoB in lipoprotein fractions**				
VLDL1 apoB (mg/dL)^b^	0.03 (0.01)	0.81 (0.70)	0.97 (0.44)	0.088
VLDL2 apoB (mg/dL)	0.13 (0.08)	1.76 (1.25)	2.75 (0.86)	0.002
IDL apoB(mg/dL)	0.68 (0.58)	1.76 (1.36)	1.71 (0.57)	0.16
LDL apoB (mg/dL)	40.6 (5.1)	51.2 (8.9)	51.5 (5.5)	0.056

*Note*: *p* values are test across groups.

^a^ Plasma LDL‐C as measured by routine assay includes IDL plus LDL‐C.

^b^ Determined by chemical analysis of isolated fractions. Plasma apoB determined by turbidimetry with differing calibration method.

**Table 2:** Lipid composition of apoB‐containing lipoproteins in ANGPTL3‐deficient and control subjects.

Moles per particle (per apoB)	Triglyceride (mol)	Cholesterol (mol)	Phospholipid (mol)
**Homozygotes (*n* = 3)**			
VLDL1^a^	7890	4140	5140
VLDL2	5790^b^	5100^b^	3780
IDL	1440	670^b^	660
LDL	400^b^	1910^b^	760
**Heterozygotes (*n* = 4)**			
VLDL1	13,100	5730	4780
VLDL2	3690	3680	2270
IDL	1160	2240	1120
LDL	210	2680	870
**Controls (*n* = 10)**			
VLDL1	15,600	4160	4620
VLDL2	3500	3280	2030
IDL	1410	2340	1280
LDL	210	2790	970

^a^ Note that these compositions are for the whole fraction as isolated from plasma. VLDL1 and VLDL2 comprise both apoB100‐ and apoB48‐containing particles with the former being predominant (based on relative concentration of these two proteins).

^b^ *p* < 0.01 for comparison across groups.

**Table 3:** ApoB48 metabolism in ANGPTL3‐deficient and control subjects.

Variable	ANGPTL3 def. homozygotes (*n* = 3)	ANGPTL3 def. heterozygotes (*n* = 4)	Controls^a^ (*n* = 9)	*p* value
**Production rates (mg/day)**				
Total (basal + PP)	394 (4)	448 (95)	406 (101)	0.689
PP chylomicron production	245 (0)	268 (47)	310 (110)	0.499
PP VLDL1 production	58 (0)	67 (19)	43 (8)	0.006
PP VLDL2 production	70 (0)	81 (23)	20 (18)	<0.001
PP total VLDL production	128 (0)	148 (41)	62 (24)	<0.001
Basal total production	22 (4)	32 (8)	33 (19)	0.570
Basal VLDL1 production	14 (4)	21 (5)	16 (10)	0.540
Basal VLDL2 production	8 (1)	11 (10)	17 (16)	0.550
**Fractional catabolic rates (pools/day)**				
Total apoB48 FCR	216 (7)	46.4 (48)	33.0 (16)	<0.001
Chylo total FCR	1005 (302)	239 (288)	139 (119)	<0.001
Chylo direct clearance	0.0 (0)	0.0 (0)	0.0 (0)	
VLDL1 FCR	712 (324)	111 (134)	58 (31)	<0.001
VLDL2 FCR	341 (33)	96 (78)	53 (23)	<0.001

Abbreviations: FCR, fractional catabolic rate; PP, post‐prandial.

^a^ Data not available for 1 control subject.

### Study approval

The study design was approved by the ethics committees of Rome, Italy (approval code # 0061/2022) and Helsinki University Central Hospital, Helsinki, Finland (Clinical Trials.gov NCT 04209816). The study was performed in accordance with the Declaration of Helsinki and the European Medicines Agency note for guidance on good clinical practice. All study participants gave a written informed consent form before any study procedures were initiated.

### Data availability

The individual‐level data generated and analysed in this study cannot be shared because the ethical approval obtained for the project does not permit disclosure of participant‐level information. Aggregated population‐level data underlying the main findings may be made available from the corresponding author upon reasonable request if the request is in accordance with the terms of the ethical permits.

## Results

Characteristics of the ANGPTL3‐deficient subjects and controls are given in Table 1. Detailed investigations of the phenotypes associated with ANGPTL3 deficiency have been reported previously [12, 13, 32]. The subject groups were matched for age and BMI, and, consistent with previous reports indicating that ANGPTL3 deficiency does not impair glucose homeostasis [12, 13], glucose and insulin levels were comparable across groups. Compared with controls, plasma ANGPTL3 was undetectable in the three homozygous subjects and was reduced by approximately 50% in the four heterozygous subjects, who were selected on the basis of low ANGPTL3 levels.

Plasma TG and cholesterol were on average lower in homozygotes compared to controls, although the difference for TG was not formally significant, probably due to the small numbers in each group. Compared to controls, apoB was 30% lower in homozygotes but not in heterozygotes (*p *= 0.009), whereas apoA1 and HDL‐C were significantly lower in homozygotes but not in heterozygotes (*p *= 0.002). These data are in accord with previous reports in larger groups of subjects heterozygous or homozygous for ANGPTL3 deficiency [12, 13, 33]. A notable further finding was the very low level of plasma apoCIII in the homozygotes, at about 15% of the concentration seen in controls (*p *< 0.001). Mean basal (fasting) levels of VLDL1‐ and VLDL2‐TG were more than 90% lower in homozygotes and reduced about 40% in heterozygotes compared to the control group (*p *= 0.037 and *p *= 0.092, respectively).

The concentration of LDL‐C was 50% lower (*p *< 0.001), whereas LDL‐TG was 68% higher in homozygotes compared to the other two groups (*p *= 0.007) (Table 1). When TG/cholesterol ratios were examined for each of the four apoB‐containing lipoprotein fractions, it was seen that homozygotes had a lower TG/chol ratio in VLDL and a significant, 2.8‐fold higher TG/chol ratio in LDL compared to heterozygotes or controls (*p *< 0.001). This ratio in IDL was also significantly higher in homozygotes (*p *= 0.015). Further analysis of lipoprotein composition (Table 2) revealed that in homozygotes LDL particles had a substantially higher molar content of TG and a lower content of cholesterol than heterozygotes or controls. IDL composition in homozygotes exhibited similar but less marked changes (more detailed compositional data are given in Table S1). The relative depletion of cholesterol in LDL particles in homozygotes has an important consequence in that LDL‐C is not a reliable reflection of LDL apoB concentration (LDL particle number). In these subjects compared to controls, the reduction in LDL apoB at 20% was only half of that seen for LDL‐C. Most of the lower plasma total apoB in homozygotes was attributable to the VLDL and IDL fractions (Table 1).

### ApoB48 metabolism in ANGPTL3‐deficient and control subjects

**Fig. 1:**
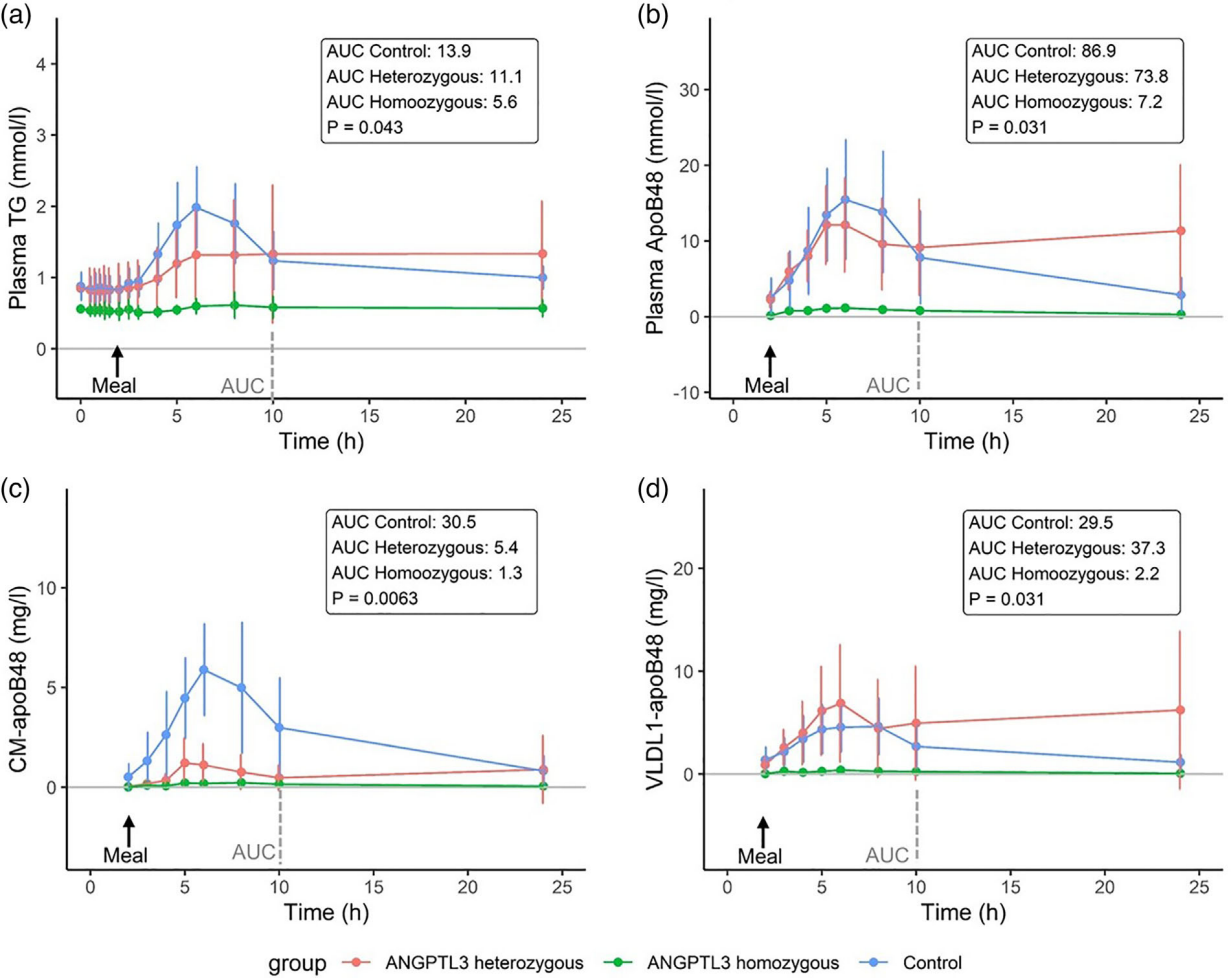
Post‐prandial responses in ANGPTL3‐deficient and control subjects. Subjects ate a standard fat‐rich meal at the 2‐h time point. Plasma TG, plasma apoB48 and the apoB48 content of the chylomicron and VLDL1 fractions were measured in serial blood samples until the 10‐h time point. Post‐prandial response was determined as the area‐under curve (AUC) in mmol/L × h or mg/dL × h over the 2–10‐h period. p values were derived by ANOVA across the three groups. The figure shows (a) plasma TG, (b) plasma apoB48, (c) chylomicron‐apoB48, and (d) VLDL1‐apoB48.

The increase in plasma TG and apoB48 levels following the fat‐rich test meal was markedly reduced in homozygotes compared to controls and heterozygotes (Fig. 1, Panels a and b) with concentrations barely rising above fasting levels (confirming results reported earlier [13, 23]). In heterozygotes, total plasma apoB48 rose and fell to the same degree as in controls. However, there appeared to be a subtle difference in that chylomicron apoB48 levels were, across the period of alimentary lipaemia (2–10 h), less than in controls (83% lower area‐under curve [AUC] in Panel c), whereas for VLDL1 apoB48, heterozygotes had slightly higher levels than in controls (26% higher AUC in Panel d).

Investigation of the kinetics of apoB48 using deuterated leucine as tracer (see Fig. S1 for the complete data set of lipoprotein concentrations and apoB48 tracer enrichment in chylomicrons, VLDL1 and VLDL2) revealed that apoB48 total production, in either the basal (pre‐meal) or post‐prandial state appeared not to differ significantly across the three groups (Table 3). We observed differences in post‐prandial apoB48 production in the VLDL density range, but these were mainly a reflection of low rates in the control group with little difference between homozygotes and heterozygotes. In homozygotes, the FCRs for total apoB48 and apoB48 in chylomicrons, VLDL1 and VLDL2 were several‐fold greater than in controls and heterozygotes, which explained the extremely low levels seen after the test meal (Fig. 1). Heterozygotes exhibited FCRs that were 1.5 to 2‐fold higher than those seen in controls (Table 3). The rapid FCR for chylomicron apoB48 in heterozygotes provides an explanation for the lower apoB48 concentrations seen in this fraction in Fig. 1 Panel c.

### ApoB100 and triglyceride metabolism in ANGPTL3‐deficient and control subjects

**Fig. 2:**
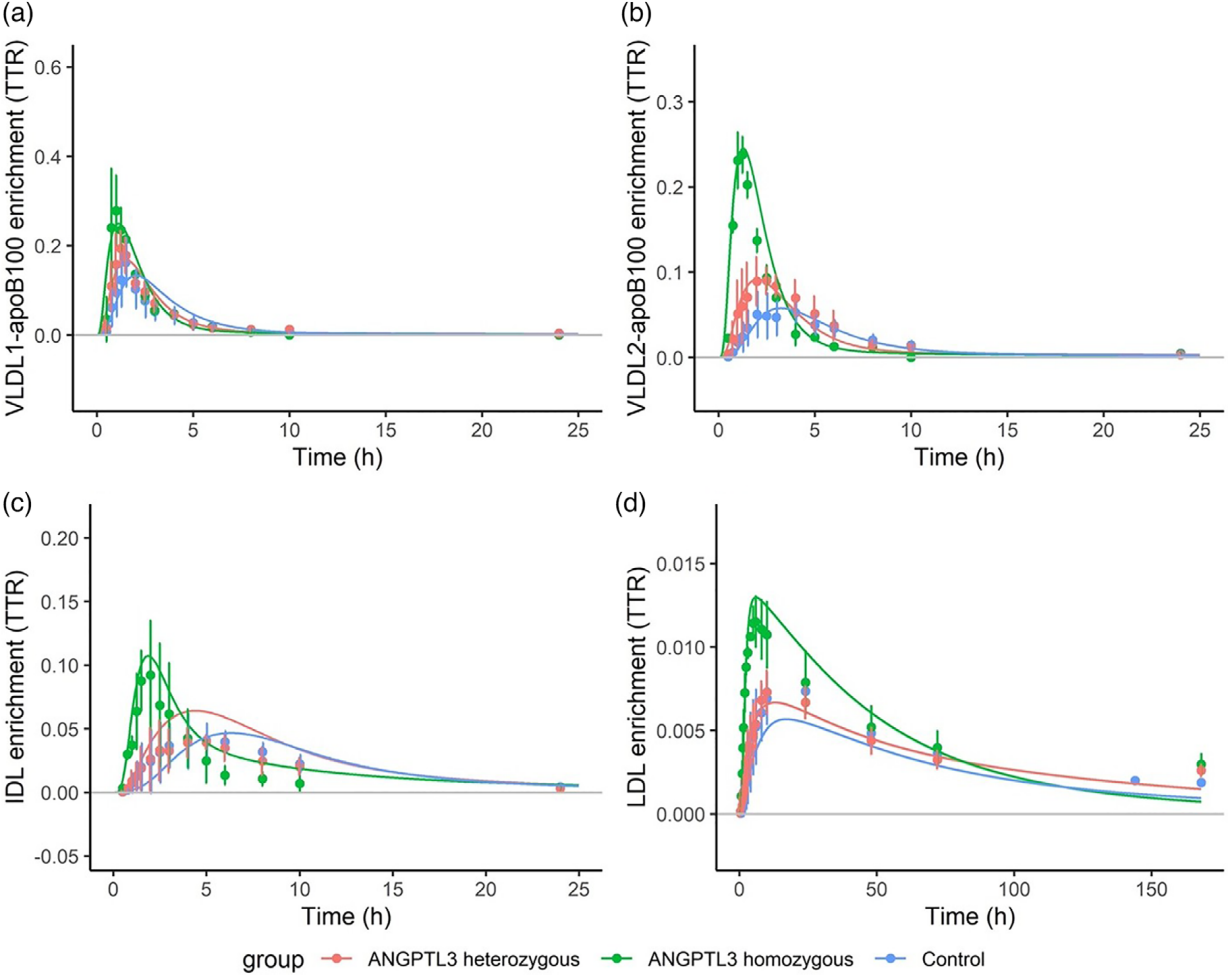
ApoB100 metabolism in ANGPTL3‐deficient and control subjects. VLDL1, VLDL2, IDL and LDL were isolated from blood samples taken from ANGPTL3 homozygotes, heterozygotes and controls at the times indicated. Enrichment of deuterated leucine in apoB was determined by mass spectrometry as described previously [26, 27]. Data points shown are group means and SD. The continuous line for each fraction is the fit from the multicompartment model described in detail in [26, 27]. The figure shows deuterated leucine enrichment curves for (a) VLDL1 apoB100, (b) VLDL2 apoB100, (c) IDL, and (d) LDL.

Deuterated leucine tracer enrichment curves for apoB100 in VLDL1, VLDL2, IDL and LDL are shown in Fig. 2. The most marked differences between groups were for homozygotes in whom tracer enrichment rose and fell very rapidly in all four lipoprotein fractions. In heterozygotes at least for VLDL1, VLDL2 and IDL apoB100, there were differences from controls with again earlier increases and decreases in tracer enrichment in these lipoprotein fractions (the full data set is shown in Fig. S1). Table 4 provides the kinetic rate constants derived from multicompartmental modelling of these data, which again need to be interpreted with appropriate caution given the small number of subjects in each group. In homozygotes, VLDL1‐ and VLDL2‐apoB100 pools were 3%–5% of the values seen in controls, whereas in heterozygotes, the mean total VLDL apoB100 pool size was 40% lower than in the control group (*p *= 0.085).

**Table 4:** ApoB100 and triglyceride metabolism in ANGPTL3‐deficient and control subjects.

Variable	ANGPTL3 def. homozygotes (*n* = 3)	ANGPTL3 def. heterozygotes (*n* = 4)	Controls (*n* = 10)	*p* value
**ApoB100 production rates (mg/day)**				
VLDL1 production	666 (36)	781 (138)	781 (123)	0.342
VLDL2 production (total)	793 (35)	691 (175)	803 (142)	0.412
Direct VLDL2 production	147 (8)	187 (19)	174 (70)	0.661
Total VLDL production	813 (30)	967 (157)	954 (139)	0.259
IDL production	516 (178)	463 (91)	503 (250)	0.936
LDL production	516 (178)	416 (58)	350 (160)	0.251
***ApoB100 pool sizes (mg)***				
VLDL1 pool	1.6 (0.3)	30 (27)	33 (12)	0.030
VLDL2 pool	5.1 (1.8)	61 (35)	119 (55)	0.006
Total VLDL pool	6.6 (1.5)	91 (58)	152 (61)	0.004
IDL pool	20 (5.8)	71 (41)	78 (38)	0.079
LDL pool	1211 (497)	1546 (283)	1621 (527)	0.451
**ApoB100 fractional catabolic rates (pools/day)**				
VLDL1 FCR	443 (104)	132 (203)	26.8 (10.7)	<0.001
VLDL1 FDC	12.4 (0.7)	10.0 (2.2)	5.03 (3.34)	0.002
VLDL1 FTR to VLDL2	430 (105)	122 (202)	21.7 (10.3)	<0.001
VLDL2 FCR	169 (56)	21.4 (25)	8.08 (3.79)	<0.001
VLDL2 FDC	58.4 (36.5)	8.4 (10.7)	3.9 (3.37)	<0.001
VLDL2 FTR to IDL	110 (74)	12.9 (14.2)	4.18 (1.34)	<0.001
Total VLDL apoB100 FCR	364 (35.2)	58.0 (57.8)	24.3 (8.01)	<0.001
Total VLDL FDC	138 (77.4)	28.1 (23.3)	12.9 (7.99)	<0.001
IDL FCR	25.3 (12.6)	7.95 (5.08)	4.71 (2.03)	<0.001
LDL FCR	0.41 (0.15)	0.27 (0.06)	0.21 (0.06)	0.006
**VLDL triglyceride kinetics**				
VLDL1 TG production (g/day)	14.5 (0.8)	23.8 (16.3)	20.2 (3.5)	0.345
VLDL2 TG production (g/day)	4.6 (0.20)	5.1 (2.0)	10.1 (3.1)	0.005
Direct VLDL2 production (g/day)	0.85 (0.05)	1.49 (0.89)	2.05 (0.57)	0.027
Total VLDL TG production (g/day)	15.3 (0.7)	25.3 (17.2)	22.3 (3.5)	0.321
VLDL1 TG FCR (pools/day)	598 (129)	183 (270)	33.6 (16.4)	<0.001
VLDL1 TG FDC (pools/day)	442 (94.7)	137 (198)	20.6 (12.5)	<0.001
VLDL1 TG FTR to VLDL2 (pools/day)	154 (34.9)	44.7 (71.7)	13.0 (7.4)	<0.001
VLDL2 TG FCR (pools/day)	281 (66)	37.8 (39.1)	19.6 (7.4)	<0.001

Abbreviations: FCR, fractional catabolic rate; FDC, fractional rate of direct catabolism (apoB100 cleared directly from the circulation); FTR, fractional transfer rate.

Production rates did not appear to differ significantly across the groups for the four lipoprotein fractions, and so the greatly reduced pool sizes for VLDL1, VLDL2 and IDL in homozygotes were the result of enhanced clearance rates in these lipoproteins. The FCR for VLDL1‐apoB100 was 17‐fold higher in homozygotes compared to controls, mainly due to a very rapid FTR to VLDL2. The fractional rate of direct VLDL1 apoB100 clearance was only moderately elevated in comparison. Likewise, the FCR for VLDL2 apoB100 was 20‐fold greater than that seen in controls due to a high FTR for the VLDL2 to IDL conversion, although VLDL2 direct clearance was also apparently greater than in controls. The total VLDL FCR and fractional rate of direct clearance were several‐fold higher in homozygotes versus controls, whereas heterozygotes exhibited FCRs that were about 2‐fold higher than in controls (Table 4).

The IDL FCR was higher in homozygotes compared to heterozygotes and controls, as was the FCR for LDL apoB100. The latter was about twice the value seen in the other two groups. The LDL apoB100 enrichment curves in homozygotes exhibited a distinct bi‐exponential decay with rapid catabolism occurring during the first 48 h followed by slower catabolism from 48 to 120 h. The latter part of the decay curve for LDL apoB100 in homozygotes was parallel to the decays seen in heterozygote and control subjects over the same period (Fig. 2 Panel d). These kinetic data indicate that LDL in homozygotes comprised two LDL subspecies with distinct metabolic characteristics.

Triglyceride kinetics in VLDL1 and VLDL2, determined using a deuterated glycerol tracer, followed the pattern seen for apoB100 (Table 4). Total TG production did not differ significantly across the three groups of subjects, although there was about a 55% lower production rate for VLDL2 TG in the homozygotes compared to controls. The FCRs and FTRs for VLDL1 and VLDL2 were several‐fold higher in homozygotes compared to controls, whereas heterozygotes had intermediate values for these rate constants.

**Fig. 3:**
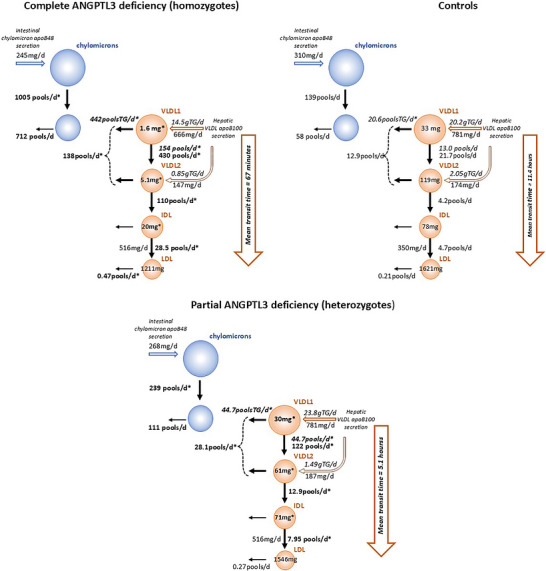
Flowchart of apoB metabolism in ANGPTL3‐deficient and control subjects. Production and clearance rates and pool sizes are taken from Tables 2 and 3. Mean transit time (in minutes or hours) was calculated for each group of subjects by summing of residence time in the circulation of VLDL1, VLDL2 and IDL. Residence time for a lipoprotein fraction is the reciprocal of the fractional catabolic rate (1/FCR). *Denotes significant difference across groups.

Fig. 3 summarizes the flow of apoB‐containing particles through the lipolytic pathway in ANGPTL3‐deficient subjects and controls. Chylomicrons and VLDL (VLDL1 and VLDL2) are produced at normal rates in homozygotes and heterozygotes, but the transit down the delipidation pathway was markedly accelerated in ANGPTL3 deficiency. Chylomicrons in homozygotes had a mean residence time (reciprocal of FCR) in the circulation of 1.4 min in homozygotes compared to 10.4 min in controls and an intermediate 6 min in heterozygotes. The mean residence times for VLDL were 10.4 and 51.2 min in homozygotes and heterozygotes, respectively, compared to 112 min in controls.

In homozygotes, the mean transit time for a VLDL1 particle to be delipidated to an LDL particle was 67 min compared to 11.4 h in the control group. Heterozygotes, also, exhibited an accelerated VLDL1 to LDL transit time (5.1 h) compared to controls (Fig. 3).

### ApoCIII and apoE metabolism in ANGPTL3‐deficient and control subjects

The deuterated leucine tracer enrichment curves for apoCIII and apoE were used to derive FCRs for these proteins (Table S2). ApoCIII FCR at 4.3 pools/day was higher in homozygotes than in heterozygotes (1.99 pools/day) and controls (1.69 pools/day) (*p* < 0.001). The apoE FCR did not differ significantly across the groups; 6.83, 4.52 and 5.48 pools/day in homozygotes, heterozygotes and controls, respectively.

## Discussion

ANGPTL3 is considered a strong candidate for targeted intervention to reduce ASCVD risk in people with elevated apoB‐containing lipoproteins. Studies of humans with naturally occurring LOF mutations in genes encoding drug targets can provide insight into the efficacy and potential adverse effects of inhibitory drugs directed at those targets [34]. In the present study, we compared subjects with an absence of ANGPTL3 in plasma (LOF homozygotes exhibiting a FHBL2 phenotype) with those having a reduced concentration of this protein (selected LOF heterozygotes exhibiting an intermediate hypolipidemic phenotype), and matched controls. What the present work adds is a comprehensive evaluation of the consequences of both complete and, for the first time, partial ANGPTL3 deficiency on apoB metabolism throughout the entire VLDL–LDL delipidation cascade.

Our key findings were that complete ANGPTL3 deficiency as seen in homozygotes gives rise to such a rapid acceleration of the normal triglyceride transport pathway that chylomicron particles circulate on average for just over a minute, whereas VLDL circulates for about 15 min instead of the usual 1–2 h and is delipidated at 10 times the normal rate into IDL and LDL. The decrease in plasma apoB is accordingly mainly attributable to the near absence of TRL and substantially lower IDL. Production rates of chylomicrons and VLDL particles did not appear to be affected by the lack of ANGPTL3. LDL in homozygotes was cholesterol‐depleted and triglyceride‐enriched. Hence, LDL‐C did not reflect the true abundance of LDL particles; the decrease in LDL‐apoB compared to controls was much less than that of LDL‐C. LDL apoB100 clearance rate was higher in ANGPTL3‐deficient homozygotes than in the other groups and exhibited complex kinetics, indicating the presence of both rapidly and slowly catabolised lipoproteins in this density range. In heterozygotes with partial ANGPTL3 deficiency, the catabolism of both chylomicron and VLDL was accelerated compared to controls but much less so than in homozygotes, whereas LDL metabolism appeared to be normal. In forming conclusions from these findings, it must be born in mind that the groups of subjects studied were by their nature small, and the ability to detect more moderate‐sized inter‐group differences was limited by this fact. That said, key aspects of the metabolic dysregulation of apoB metabolism in ANGPTL3 deficiency were clearly revealed in this investigation and have implications for therapeutic interventions, especially for those agents directed at inhibiting ANGPTL3 that markedly reduce but do not eliminate the protein from the circulation.

In light of the dramatic acceleration of lipolysis in subjects homozygous for ANGPTL3 deficiency, the question arises as to how the tissues of these individuals cope with the accelerated flux of FFA released in capillary beds. One might imagine that such a metabolic change could lead to an expansion of adipose depots. However, our previous studies conducted using nuclear magnetic resonance techniques showed no changes in the amount of visceral, subcutaneous, abdominal or intermuscular adipose tissue [25]. Alternatively, it could be hypothesized that the notable increase in LPL activity linked to the loss of ANGPTL3 inhibition may affect tissues with oxidative activity to a greater extent. This possibility is partially supported by the observation that subjects with complete ANGPTL3 deficiency show higher levels of ketone bodies (such as β‐hydroxybutyrate) both in the fasting and post‐prandial states [35]. As ketogenesis is elevated when the influx of fatty acids into the liver is increased [36], this observation may indicate that complete ANGPTL3 deficiency is accompanied by an increased hepatic utilization of fatty acids derived from the lipolysis of TRL. Whether this phenomenon also occurs in muscles remains to be determined.

The impact on post‐prandial response to a fat load test has been investigated in ANGPTL3‐deficient homozygotes previously with results consistent with those seen here. Tikkanen et al. [35] and Fappi et al. [23] found that in homozygotes there is almost no increase in plasma and chylomicron TG and apoB48 following a fat‐rich meal. In line with the kinetic study of Fappi et al. [23], we noted no difference between ANGPTL3‐absent homozygotes and controls in total apoB48 production and a greatly increased apoB48 FCR with very short residence times for chylomicron particles in the former group. Our data also agree with the earlier investigation regarding the several‐fold increase in VLDL FCR with about a 10‐fold decrease in residence time for TRL containing apoB100. Where the reports differ is that Fappi et al. [23] found an approximate 50% reduction in VLDL particle (TRL‐apoB100) production rate in their *ANGPTL3* double heterozygotes, whereas in the present study, we observed near normal rates. There are a number of possible reasons for this discrepancy. Subject numbers were small with each study, including only three individuals with complete deficiency; the genetic defect in *ANGPTL3* differed, and in the Fappi et al. study, two of the three subjects were diabetic [23]. A further complication is that the comparator control groups differed in lipid profile. Our controls had a low plasma TG of 0.85 mmol/L compared to a plasma TG of 1.27 mmol/L in Fappi et al. [23]. As plasma TG is correlated positively with hepatic VLDL secretion rate in the normal population, this may explain at least some of the apparent discordancy in the results [37, 38, 39]. In the study of Reeskamp et al. [9], reporting the impact of the ANGPTL3 blocking antibody evinacumab on lipoprotein kinetics in homozygous FH subjects, there was no overall effect on VLDL‐apoB production, no change in LDL‐apoB production rates and a marked increase in the FCRs of IDL and LDL‐apoB. We too saw no difference in LDL production rates, and the magnitude of increase in IDL and LDL FCRs (6‐ and 2‐fold, respectively) in ANGPTL3‐deficient homozygotes compared to controls was similar to that of ANGPTL3 inhibition with evinacumab in homozygous FH [9].

It has been suggested that the response to ANGPTL3 deficiency or inhibition with an antibody or RNA agent may differ according to a recipient's hyperlipidaemic genotype/phenotype [10]. The reduction in LDL‐C in homozygous FH subjects is substantial and occurs in those who are double null with an absence of LDL receptors and those who are receptor defective with residual activity. In the study of Reeskamp et al., the former showed a reduction in VLDL‐apoB production, whereas in the latter, there was no change in this parameter (although there were only two subjects in each group). It is unclear at present how the status of the LDL receptor pathway affects response to ANGPTL3 blocking, but animal models also support such an interaction (White O, Aligabi Z, Burks KH, et al. Endothelial lipase facilitates low‐density lipoprotein (LDL) uptake in LDL receptor deficiency by a heparan sulfate proteoglycan‐dependent mechanism. Although overall VLDL‐TG secretion in our study was comparable to control values, we did see a reduction in VLDL2‐TG secretion in heterozygous and homozygous subjects even though the rate of direct VLDL2‐apoB100 production was comparable to controls. Thus, we cannot exclude a subtle effect of ANGPTL3 on VLDL assembly and secretion, possibly affecting mainly smaller VLDL particles. Finally, it was noted that post‐prandial VLDL1‐ and VLDL2‐apoB48 production rates were higher in both ANGPTL3 LOF heterozygotes and homozygotes compared to controls. This may be a feature of intestinal lipoprotein synthesis during fat absorption in these subjects, but also given that overall apoB48 production was comparable to normal (in the present study and [23]), it may be the result of the very rapid lipolysis of chylomicrons as they enter the bloodstream so that the particles appear in the VLDL density range almost immediately.

In interpreting our findings, it is worth noting that plasma apoCIII was markedly reduced in the homozygotes to levels 50% lower than those we observed in heterozygous *APOC3* LOF carriers [40]. This accords with the observation that plasma apoCIII is reduced markedly by evinacumab treatment [41]. As apoCIII is a major regulator of TRL metabolism, the greatly accelerated lipolysis rates seen in homozygotes may be attributed to the combination of an absence of ANGPTL3 and reduced apoCIII levels. Drawing on previous kinetic data from *APOC3* LOF heterozygotes, who exhibited a 3.7‐fold higher VLDL1 FCR than controls [40], we can cautiously estimate that roughly 20% of the 17‐fold increase in VLDL1 clearance observed in our ANGPTL3 homozygotes might be attributable to the concomitant lowering of apoCIII.

The decreased apoCIII concentration in homozygotes was linked to a rapid clearance rate for the protein, which is likely due to the very low concentrations of TRL and the subnormal HDL level; indeed, most apoCIII in the circulation is carried on these two lipoproteins, and when they are depleted, apoCIII is cleared at an accelerated rate. Thus, when a chylomicron or VLDL particle enters the bloodstream of homozygous subjects, it is likely to be acted on by LPL that is no longer regulated (inhibited) by apoCIII and ANGPTL3. The very rapid lipolysis that ensues is a reflection of the high capacity of the lipase system, and within minutes, as opposed to hours, end products ‘remnant particles’ are formed. Normally, TRL remnants acquire cholesteryl ester by transfer from HDL via CETP, become cholesterol‐enriched and attain the size of small VLDL or IDL [42], but when lipolysis is so rapid, remnant‐like particles will likely still have a TG‐rich core (having had little opportunity to gain cholesteryl ester) and may end up mainly in the IDL/LDL size range. One interpretation of the altered composition and kinetics of LDL—the presence of two metabolically distinct species—in our homozygotes is that this fraction contains abnormal TG‐rich remnant‐like lipoproteins that are cleared more quickly than normal LDL as well as LDL particles of normal composition and metabolic properties. In heterozygotes, the 5‐h transit time from VLDL to LDL is similar to that seen in *APOC3* LOF heterozygotes, and, as in that condition, LDL exhibits normal composition and metabolic behaviour. An additional or alternative explanation for enhanced LDL catabolism in ANGPTL3‐absent homozygotes entails the action of EL, which is normally inhibited by ANGPTL3. This enzyme can modify the lipid content of LDL particles and thereby open up a route of LDL clearance in hepatocytes that is independent of the classic LDL receptor‐mediated pathway [10]. This mechanism may account for the finding that LDL is reduced by evinacumab in receptor‐deficient homozygous FH subjects [9].

The consequences for atherogenesis of these substantial changes in the structure and metabolic properties of apoB‐containing lipoproteins in ANGPTL3 deficiency are at the moment unclear. There is genetic evidence that ANGPTL3 deficiency is associated with reduced ASCVD risk, but it is not as consistent as for other LPL regulatory proteins, such as apoCIII [16, 18].

The effects of complete and partial ANGPTL3 deficiency on apoB metabolism can be compared with the pharmacodynamic action of agents that block ANGPTL3 synthesis or inhibit its action. Zodasiran, an siRNA directed at the *ANGPTL3* gene, in hypertriglyceridaemic subjects lowered plasma ANGPTL3 about 70% at the 100/200 mg dose. Plasma TG was reduced 57%–63%, remnant cholesterol by 76%–80%, LDL‐C by 14%–20%, HDL‐C by 22%–24% and apoB by 15%–22%. The achieved per cent ANGPTL3 decrease on zodasiran was midway between the reduced levels seen in heterozygotes (50% lower) and homozygotes (100% lower) versus controls in the present study, and so the effect of the drug on apoB metabolism may be modelled as intermediate between the patterns seen in our LOF heterozygotes and homozygotes. It should be noted, however, that our heterozygotes were specifically selected for low ANGPTL3 concentrations to better model this pharmacological reduction, and therefore, their kinetic profile may represent a best‐case scenario that overestimates the expected effect in an unselected heterozygous population. The decrease in apoB is likely to be largely in the TRL fraction. Using a TRL cholesterol/apoB ratio of 2.2 [43], the decrease of 37.6 mg/dL in remnant cholesterol on zodasiran would be accompanied by a fall of about 17 mg/dL in TRL apoB, out of the total apoB drop of approximately 18 mg/dL. Further, taking into account the compositional perturbation in LDL we observed in homozygotes, the modest decrease in LDL‐C on zodasiran may not reflect a reduction in LDL particle number (the drop in LDL apoB is predicted to be small) but rather a compositional change in the lipoprotein. Results from trials of evinacumab in hypertriglyceridemic subjects report a similar pattern of plasma lipid changes [44]. From the results of the present investigation, it follows that *ANGPTL3* silencing or ANGPTL3 inhibition with antibodies is likely to impact mainly chylomicron/apoB48 metabolism, the lipolysis rate of VLDL1 and VLDL2, and IDL catabolism but have only modest effect on LDL‐apoB100 kinetics in moderately hypertriglyceridemic individuals.

There are limitations in the present study. Most evident is the small number of ANGPTL3‐deficient subjects available for kinetic investigation. This hampers formal statistical comparisons but as the effect sizes are large and the plasma lipid profiles in ANGPTL3‐deficient subjects in line with those described earlier using larger subject groups, there is reason to have confidence in the findings.

A number of questions still remain regarding aspects of apoB metabolism in ANGPTL3 deficiency, whether it is caused by inherited LOF variants or ANGPTL3 blocking drugs. First, what is the structure of lipoprotein particles in the LDL density range—what is the composition, physical properties and atherogenic potential of the metabolically distinct species that are present. Second, does ANGPTL3 inhibition affect VLDL production in the liver. As discrepant results have been obtained in small subject groups, this issue needs addressed in a much larger kinetic study of ANGPTL3 inhibition. Third, what are the consequences for whole body triglyceride and fatty acid metabolism of the very rapid lipolysis of TRL. Can TG delivery be directed as required to the appropriate tissues.

In conclusion, we found that complete ANGPTL3 deficiency is accompanied by a secondary, substantial reduction in apoCIII. The loss of these two key regulators of LPL led to a pronounced acceleration of the VLDL–LDL delipidation cascade. LDL had an altered composition and metabolism in homozygotes which needs to be taken into account when interpreting lipid changes on drugs that inhibit ANGPTL3. Our finding in partial ANGPTL3 deficiency in heterozygotes affected TRL metabolism but not LDL which is noteworthy. It is likely that targeting ANGPTL3 with drugs will give an outcome in terms of apoB‐containing lipoprotein kinetics that lies somewhere between what we found in the heterozygotes and in homozygotes. Present understanding of the implications for ASCVD prevention of ANGPTL3 inhibition will depend on the degree of TRL/remnant lowering and the unknown consequences of perturbing the properties of LDL.

## Author contributions

Marcello Arca, Marja‐Riitta Taskinen, Chris J. Packard and Jan Borén contributed to conception and design. Elias Björnson, Laura D'Erasmo, Alessia Di Costanzo, Simone Bini, Ilenia Minicocci, Daniele Tramontano, Stella Covino, Giuseppe Ciarlo, Manuela Lombardi, Sanni Söderlund, Niina Matikainen, Linda Andersson, Marcello Arca to the acquisition of data or analysis, and Marcello Arca, Elias Björnson, Marja‐Riitta Taskinen, Chris J. Packard and Jan Borén to the interpretation of data. Marcello Arca, Elias Björnson, Marja‐Riitta Taskinen, Chris J. Packard and Jan Borén drafted the original and revised manuscripts and all authors approved the final approval of the version to be published.

## Conflict of interest statement

Marcello Arca has received research grant support and/or honoraria, consulting or lecturing fees from Alfasigma, Amarin, Amgen, Amryt, Aegerion, Akcea/Ionis, Arrowhead, Daiichi Sankyo, Chiesi, Lilly, Novartis, Pfizer, Regeneron, Sanofi, SOBI and Ultragenix. Elias Björnson declares honoraria/consulting fees for Arrowhead Pharmaceuticals, Novartis and Ribocure. Laura D'Erasmo has received honoria/consulting fees and grant supports from AuroraBiopharma, Amarin, Amgen, Amryt Pharmaceutical, Bayer, Chiesi, Daiichi‐Sankyo, Novartis, Sandoz, SOBI and Ultragenyx. Niina Matikainen has received consulting fees or advisory board honoraria from Amgen, Novo Nordisk, Organon. Marja‐Riitta Taskinen has received consulting fees or advisory board honoraria from Amgen, Novo Nordisk, Eli Lilly and Sanofi. Chris J. Packard declares honoraria/consulting fees from, Amarin, Arrowhead, Dalcor, Pfizer and Response Therapeutics. Jan Borén declares honoraria/consulting fees from Novartis, Novo Nordisk, Akcea, Amgen, Ribocure and Pfizer. The remaining authors declare no conflicts of interest.

## Funding information

The 2021 Global ANGPTL3 ASPIRE Cardiovascular Competitive Grant Program, the Swedish Heart‐Lung Foundation, the Swedish Research Council, and Swedish governmental funding of clinical research (ALF).

## Supporting information




**Table S1**: Composition of apoB‐containing lipoproteins (mg/mg) in fasting state.
**Table S2**: ApoCIII and apoE fractional clearance rates in ANGPTL3‐deficient and control subjects
**Figure S1**: ApoB concentrations and tracer enrichment in ANGPTL3‐deficient (homozygotes and heterozygotes) and control subjects.

## Data Availability

The data that support the findings of this study are available on request from the corresponding author. The data are not publicly available due to privacy or ethical restrictions.

## References

[1] Wu SA , Kersten S , Qi L Lipoprotein lipase and its regulators: an unfolding story. Trends Endocrinol Metab. 2021;32:48–61. 33277156 10.1016/j.tem.2020.11.005 PMC8627828

[2] Kersten S New insights into angiopoietin‐like proteins in lipid metabolism and cardiovascular disease risk. Curr Opin Lipidol. 2019;30:205–211. 30893111 10.1097/MOL.0000000000000600

[3] Kersten S Angiopoietin‐like 3 in lipoprotein metabolism. Nat Rev Endocrinol. 2017;13:731–739. 28984319 10.1038/nrendo.2017.119

[4] Fujimoto K , Koishi R , Shimizugawa T , Ando Y Angptl3‐null mice show low plasma lipid concentrations by enhanced lipoprotein lipase activity. Exp Anim. 2006;55:27–34. 16508209 10.1538/expanim.55.27

[5] Sylvers‐Davie KL , Davies BSJ Regulation of lipoprotein metabolism by ANGPTL3, ANGPTL4, and ANGPTL8. Am J Physiol Endocrinol Metab. 2021;321:E493–E508. 34338039 10.1152/ajpendo.00195.2021 PMC8560382

[6] Dijk W , Kersten S Regulation of lipid metabolism by angiopoietin‐like proteins. Curr Opin Lipidol. 2016;27:249–256. 27023631 10.1097/MOL.0000000000000290

[7] Brandts J , Ray KK Novel and future lipid‐modulating therapies for the prevention of cardiovascular disease. Nat Rev Cardiol. 2023;20:600–616. 37055535 10.1038/s41569-023-00860-8

[8] Ginsberg HN , Goldberg IJ Broadening the scope of dyslipidemia therapy by targeting APOC3 (apolipoprotein C3) and ANGPTL3 (angiopoietin‐like protein 3). Arterioscler Thromb Vasc Biol. 2023;43:388–398. 36579649 10.1161/ATVBAHA.122.317966 PMC9975058

[9] Reeskamp LF , Millar JS , Wu L , Jansen H , van Harskamp D , Schierbeek H , et al. ANGPTL3 inhibition with evinacumab results in faster clearance of IDL and LDL apoB in patients with homozygous familial hypercholesterolemia‐brief report. Arterioscler Thromb Vasc Biol. 2021;41:1753–1759. 33691480 10.1161/ATVBAHA.120.315204 PMC8057526

[10] Adam RC , Mintah IJ , Alexa‐Braun CA , Shihanian LM , Lee JS , Banerjee P , et al. Angiopoietin‐like protein 3 governs LDL‐cholesterol levels through endothelial lipase‐dependent VLDL clearance. J Lipid Res. 2020;61:1271–1286. 32646941 10.1194/jlr.RA120000888 PMC7469887

[11] Pisciotta L , Favari E , Magnolo L , Simonelli S , Adorni MP , Sallo R , et al. Characterization of three kindreds with familial combined hypolipidemia caused by loss‐of‐function mutations of ANGPTL3. Circ Cardiovasc Genet. 2012;5:42–50. 22062970 10.1161/CIRCGENETICS.111.960674

[12] Minicocci I , Montali A , Robciuc MR , Quagliarini F , Censi V , Labbadia G , et al. Mutations in the ANGPTL3 gene and familial combined hypolipidemia: a clinical and biochemical characterization. J Clin Endocrinol Metab. 2012;97:E1266–E1275. 22659251 10.1210/jc.2012-1298 PMC5393441

[13] Minicocci I , Tikka A , Poggiogalle E , Metso J , Montali A , Ceci F , et al. Effects of angiopoietin‐like protein 3 deficiency on postprandial lipid and lipoprotein metabolism. J Lipid Res. 2016;57:1097–1107. 27040449 10.1194/jlr.P066183 PMC4878193

[14] Robciuc MR , Maranghi M , Lahikainen A , Rader D , Bensadoun A , Öörni K , et al. Angptl3 deficiency is associated with increased insulin sensitivity, lipoprotein lipase activity, and decreased serum free fatty acids. Arterioscler Thromb Vasc Biol. 2013;33:1706–1713. 23661675 10.1161/ATVBAHA.113.301397

[15] Tikka A , Soronen J , Laurila PP , Metso J , Ehnholm C , Jauhiainen M Silencing of ANGPTL 3 (angiopoietin‐like protein 3) in human hepatocytes results in decreased expression of gluconeogenic genes and reduced triacylglycerol‐rich VLDL secretion upon insulin stimulation. Biosci Rep. 2014;34:e00160. 25495645 10.1042/BSR20140115 PMC4266921

[16] Landfors F , Henneman P , Chorell E , Nilsson SK , Kersten S Drug‐target Mendelian randomization analysis supports lowering plasma ANGPTL3, ANGPTL4, and APOC3 levels as strategies for reducing cardiovascular disease risk. Eur Heart J Open. 2024;4:oeae035. 38895109 10.1093/ehjopen/oeae035 PMC11182694

[17] Reyes‐Soffer G , Sztalryd C , Horenstein RB , Holleran S , Matveyenko A , Thomas T , et al. Effects of APOC3 heterozygous deficiency on plasma lipid and lipoprotein metabolism. Arterioscler Thromb Vasc Biol. 2019;39:63–72. 30580564 10.1161/ATVBAHA.118.311476 PMC6309928

[18] Stitziel NO , Khera AV , Wang X , Bierhals AJ , Vourakis AC , Sperry AE , et al. ANGPTL3 deficiency and protection against coronary artery disease. J Am Coll Cardiol. 2017;69:2054–2063. 28385496 10.1016/j.jacc.2017.02.030 PMC5404817

[19] Dewey FE , Gusarova V , Dunbar RL , O'Dushlaine C , Schurmann C , Gottesman O , et al. Genetic and pharmacologic inactivation of ANGPTL3 and cardiovascular disease. N Engl J Med. 2017;377:211–221. 28538136 10.1056/NEJMoa1612790 PMC5800308

[20] Chan DC , Watts GF Inhibition of the ANGPTL3/8 complex for the prevention and treatment of atherosclerotic cardiovascular disease. Curr Atheroscler Rep. 2024;27:6. 39565562 10.1007/s11883-024-01254-y

[21] Bini S , Tramontano D , Minicocci I , Di Costanzo A , Tambaro F , D'Erasmo L , et al. How ANGPTL3 inhibition will help our clinical practice? Curr Atheroscler Rep. 2023;25:19–29. 36607583 10.1007/s11883-022-01076-w

[22] Laffin LJ , Nicholls SJ , Scott RS , Clifton PM , Baker J , Sarraju A , et al. Phase 1 trial of CRISPR‐Cas9 gene editing targeting ANGPTL3. N Engl J Med. 2025;393:2119–2130. 41211945 10.1056/NEJMoa2511778

[23] Fappi A , Patterson BW , Burks KH , Davidson NO , Vaisar T , Kanter JE , et al. Effect of complete, lifelong ANGPTL3 deficiency on triglyceride‐rich lipoprotein kinetics. Cell Rep Med. 2025;6:102152. 40446802 10.1016/j.xcrm.2025.102152 PMC12208341

[24] Pennisi G , Maurotti S , Ciociola E , Jamialahmadi O , Bertolazzi G , Mirarchi A , et al. ANGPTL3 downregulation increases intracellular lipids by reducing energy utilization. Arterioscler Thromb Vasc Biol. 2024;44:1086–1097. 38385290 10.1161/ATVBAHA.123.319789

[25] D'Erasmo L , Di Martino M , Neufeld T , Fraum TJ , Kang CJ , Burks KH , et al. ANGPTL3 deficiency and risk of hepatic steatosis. Circulation. 2023;148:1479–1489. 37712257 10.1161/CIRCULATIONAHA.123.065866 PMC10805521

[26] Björnson E , Packard CJ , Adiels M , Andersson L , Matikainen N , Söderlund S , et al. Investigation of human apoB48 metabolism using a new, integrated non‐steady‐state model of apoB48 and apoB100 kinetics. J Intern Med. 2019;285:562–577. 30779243 10.1111/joim.12877 PMC6849847

[27] Björnson E , Packard CJ , Adiels M , Andersson L , Matikainen N , Söderlund S , et al. Apolipoprotein B48 metabolism in chylomicrons and very low‐density lipoproteins and its role in triglyceride transport in normo‐ and hypertriglyceridemic human subjects. J Intern Med. 2020;288:422–438. 31846520 10.1111/joim.13017

[28] Matikainen N , Mänttäri S , Schweizer A , Ulvestad A , Mills D , Dunning BE , et al. Vildagliptin therapy reduces postprandial intestinal triglyceride‐rich lipoprotein particles in patients with type 2 diabetes. Diabetologia. 2006;49:2049–2057. 16816950 10.1007/s00125-006-0340-2

[29] Taskinen M‐R , Björnson E , Kahri J , Söderlund S , Matikainen N , Porthan K , et al. Effects of evolocumab on the postprandial kinetics of Apo (apolipoprotein) B100‐ and B48‐containing lipoproteins in subjects with type 2 diabetes. Arterioscler Thromb Vasc Biol. 2021;41:962–975. 33356392 10.1161/ATVBAHA.120.315446

[30] Adiels M , Taskinen M‐R , Björnson E , Andersson L , Matikainen N , Söderlund S , et al. Role of apolipoprotein C‐III overproduction in diabetic dyslipidaemia. Diabetes Obes Metab. 2019;21:1861–1870. 30972934 10.1111/dom.13744

[31] Barrett PH , Bell BM , Cobelli C , Golde H , Schumitzky A , Vicini P , et al. SAAM II: simulation, analysis, and modeling software for tracer and pharmacokinetic studies. Metabolism. 1998;47:484–492. 9550550 10.1016/s0026-0495(98)90064-6

[32] Bea AM , Franco‐Marín E , Marco‐Benedí V , Jarauta E , Gracia‐Rubio I , Cenarro A , et al. ANGPTL3 gene variants in subjects with familial combined hyperlipidemia. Sci Rep. 2021;11:7002. 33772079 10.1038/s41598-021-86384-y PMC7997994

[33] Tarugi P , Bertolini S , Calandra S . Angiopoietin‐like protein 3 (ANGPTL3) deficiency and familial combined hypolipidemia. J Biomed Res. 2019;33:73–81. 29752428 10.7555/JBR.32.20170114 PMC6477171

[34] Young EP , Stitziel NO Capitalizing on insights from human genetics to identify novel therapeutic targets for coronary artery disease. Annu Rev Med. 2019;70:19–32. 30355262 10.1146/annurev-med-041717-085853

[35] Tikkanen E , Minicocci I , Hällfors J , Di Costanzo A , D'Erasmo L , Poggiogalle E , et al. Metabolomic signature of angiopoietin‐like protein 3 deficiency in fasting and postprandial state. Arterioscler Thromb Vasc Biol. 2019;39:665–674. 30816800 10.1161/ATVBAHA.118.312021

[36] Nosadini R , Avogaro A , Trevisan R , et al. Acetoacetate and 3‐hydroxybutyrate kinetics in obese and insulin‐dependent diabetic humans. Am J Physiol. 1985;248:R611–R620. 3922234 10.1152/ajpregu.1985.248.5.R611

[37] Mittendorfer B , Yoshino M , Patterson BW , Klein S VLDL triglyceride kinetics in lean, overweight, and obese men and women. J Clin Endocrinol Metab. 2016;101:4151–4160. 27588438 10.1210/jc.2016-1500 PMC5095238

[38] Borén J , Taskinen M , Packard CJ Biosynthesis and metabolism of ApoB‐containing lipoproteins. Annu Rev Nutr. 2024;44:179–204. 38635875 10.1146/annurev-nutr-062222-020716

[39] Borén J , Taskinen M , Björnson E , Packard CJ Metabolism of triglyceride‐rich lipoproteins in health and dyslipidaemia. Nat Rev Cardiol. 2022;19:577–592. 35318466 10.1038/s41569-022-00676-y

[40] Taskinen M‐R , Björnson E , Matikainen N , Söderlund S , Rämö J , Ainola M‐M , et al. Postprandial metabolism of apolipoproteins B48, B100, C‐III, and E in humans with APOC3 loss‐of‐function mutations. JCI Insight. 2022;7:e160607. 36040803 10.1172/jci.insight.160607 PMC9675484

[41] Raal FJ , Rosenson RS , Reeskamp LF , Hovingh GK , Kastelein JJP , Rubba P , et al. Evinacumab for homozygous familial hypercholesterolemia. N Engl J Med. 2020;383:711–720. 32813947 10.1056/NEJMoa2004215

[42] Barter PJ , Brewer HB Jr. , Chapman MJ , Hennekens CH , Rader DJ , Tall AR . Cholesteryl ester transfer protein: a novel target for raising HDL and inhibiting atherosclerosis. Arterioscler Thromb Vasc Biol. 2003;23:160–167. 12588754 10.1161/01.atv.0000054658.91146.64

[43] Sacks FM , Alaupovic P , Moye LA , Cole TG , Sussex B , Stampfer MJ , et al. VLDL, apolipoproteins B, CIII, and E, and risk of recurrent coronary events in the cholesterol and recurrent events (CARE) trial. Circulation. 2000;102:1886–1892. 11034934 10.1161/01.cir.102.16.1886

[44] Rosenson RS , Gaudet D , Ballantyne CM , Baum SJ , Bergeron J , Kershaw EE , et al. Evinacumab in severe hypertriglyceridemia with or without lipoprotein lipase pathway mutations: a phase 2 randomized trial. Nat Med. 2023;29:729–737. 36879129 10.1038/s41591-023-02222-w PMC10033404

