# Supplementary material for: Angiopoietin‐like protein 3 complete and partial deficiency markedly accelerates apolipoprotein B48 and B100 metabolism in triglyceride‐rich lipoproteins in humans
Source: J Intern Med. 2026 Jun 11;300(3):312–28. doi: 10.1111/joim.70124 (PMC13429006; doi:10.1111/joim.70124)

SUPPLEMENTAL FILE

ANGPTL3 complete and partial deficiency markedly accelerates apolipoprotein B48 and B100 metabolism in triglyceride-rich lipoproteins in humans

Running title: ApoB metabolism in ANGPTL3 deficiency

Marcello Arca[1], Elias Björnson[2], Laura D'Erasmo[1], Alessia Di Costanzo[1], Simone Bini[1], Ilenia Minicocci[1], Daniele Tramontano[1], Stella Covino[1], Giuseppe Ciarlo[3], Manuela Lombardi[3], Sanni Söderlund[4,5], Niina Matikainen[4,5], Linda Andersson[2], Martin Adiels[2], Marja-Riitta Taskinen[4]*, Chris J Packard[6]* and Jan Borén[2]*

*shared last authors

[1]Department of Translational and Precision Medicine, University of Rome Sapienza, Rome, Italy; [2]Department of Molecular and Clinical Medicine, Institute of Medicine, University of Gothenburg, Gothenburg, Sweden; [3]San Giovanni di Dio, Fondi Hospital, ASL Latina, Italy; [4]Research Programs Unit, Clinical and Molecular Medicine, University of Helsinki, Helsinki, Finland; [5]Endocrinology, Abdominal Center, Helsinki University Hospital, Helsinki, Finland; [6]Institute of Cardiovascular and Medical Sciences, University of Glasgow, Glasgow, UK

Supplementary Table 1
Composition of apoB-containing lipoproteins (mg/mg) in fasting state.

	ANGPTL3 homozygotes N=3	ANGPTL3 heterozygotes N=4	CONTROLS N=10	p
**Chylomicrons**				
Triglycerides (mean (SD))	41.68 (23.24)	67.85 (3.39)	73.50 (8.16)	0.002
Cholesterol (mean (SD))	21.63 (22.42)	11.39 (1.45)	6.09 (2.68)	0.052
Phospholipids (mean (SD))	36.41 (39.06)	18.25 (3.76)	14.46 (5.80)	0.137
Total protein (mean (SD))	0.27 (0.28)	2.51 (1.84)	5.96 (4.81)	0.092
**VLDL1**				
Triglycerides (mean (SD))	40.01 (21.95)	60.72 (2.18)	65.60 (3.05)	0.002
Cholesterol (mean (SD))	28.08 (30.11)	11.96 (0.92)	6.98 (1.47)	0.045
Phospholipids (mean (SD))	29.38 (6.20)	19.68 (1.83)	16.63 (0.79)	<0.001
Total protein (mean (SD))	2.53 (2.06)	7.64 (1.15)	10.79 (2.56)	<0.001
**VLDL2**				
Triglycerides (mean (SD))	43.54 (1.60)	42.69 (4.08)	43.83 (4.09)	0.883
Cholesterol (mean (SD))	17.63 (2.25)	19.87 (2.65)	18.49 (3.02)	0.574
Phospholipids (mean (SD))	28.17 (1.88)	24.28 (1.89)	23.27 (1.03)	<0.001
Total protein (mean (SD))	10.66 (1.33)	13.15 (1.01)	14.41 (1.84)	0.012
**IDL**				
Triglycerides (mean (SD))	28.94 (5.08)	25.39 (4.50)	25.62 (6.74)	0.689
Cholesterol (mean (SD))	8.09 (5.36)	21.13 (4.68)	21.59 (5.04)	0.003
Phospholipids (mean (SD))	13.50 (6.80)	21.15 (2.35)	22.87 (2.21)	0.003
Total protein (mean (SD))	49.47 (11.22)	32.33 (5.46)	29.92 (4.60)	0.001
**LDL**				
Triglycerides (mean (SD))	15.26 (1.76)	6.94 (0.69)	6.48 (1.43)	<0.001
Cholesterol (mean (SD))	32.18 (2.43)	40.33 (1.45)	40.00 (1.42)	<0.001
Phospholipids (mean (SD))	25.24 (1.12)	25.98 (0.19)	27.62 (0.71)	<0.001
Total protein (mean (SD))	27.32 (1.67)	26.75 (1.75)	25.91 (1.59)	0.389

Supplementary Table 2

ApoCIII and apoE fractional clearance rates in ANGPTL3 deficient and control subjects

Fractional catabolic rate	ANGPTL3 homozygotes N=3	ANGPTL3 heterozygotes N=4	CONTROLS N=9	p
ApoCIII (mean (SD))	4.3 (1.1)	2.0 (0.3)	1.7 (0.6)	<0.001
ApoE (mean (SD))	6.8 (3.4)	4.5 (1.7)	5.5 (2.1)	0.424

There were technical challenges in determining the kinetics of apoCIII in ANGPTL3 deficient subjects due to the very low plasma levels, The FCRs presented above were derived from the mono-exponential component of the decay curve for tracer enrichments for each protein. All subject data was analysed using the same procedure.

Supplementary Figure 1

ApoB concentrations and tracer enrichment in ANGPTL3 deficient (homozygotes and heterozygotes) and control subjects.

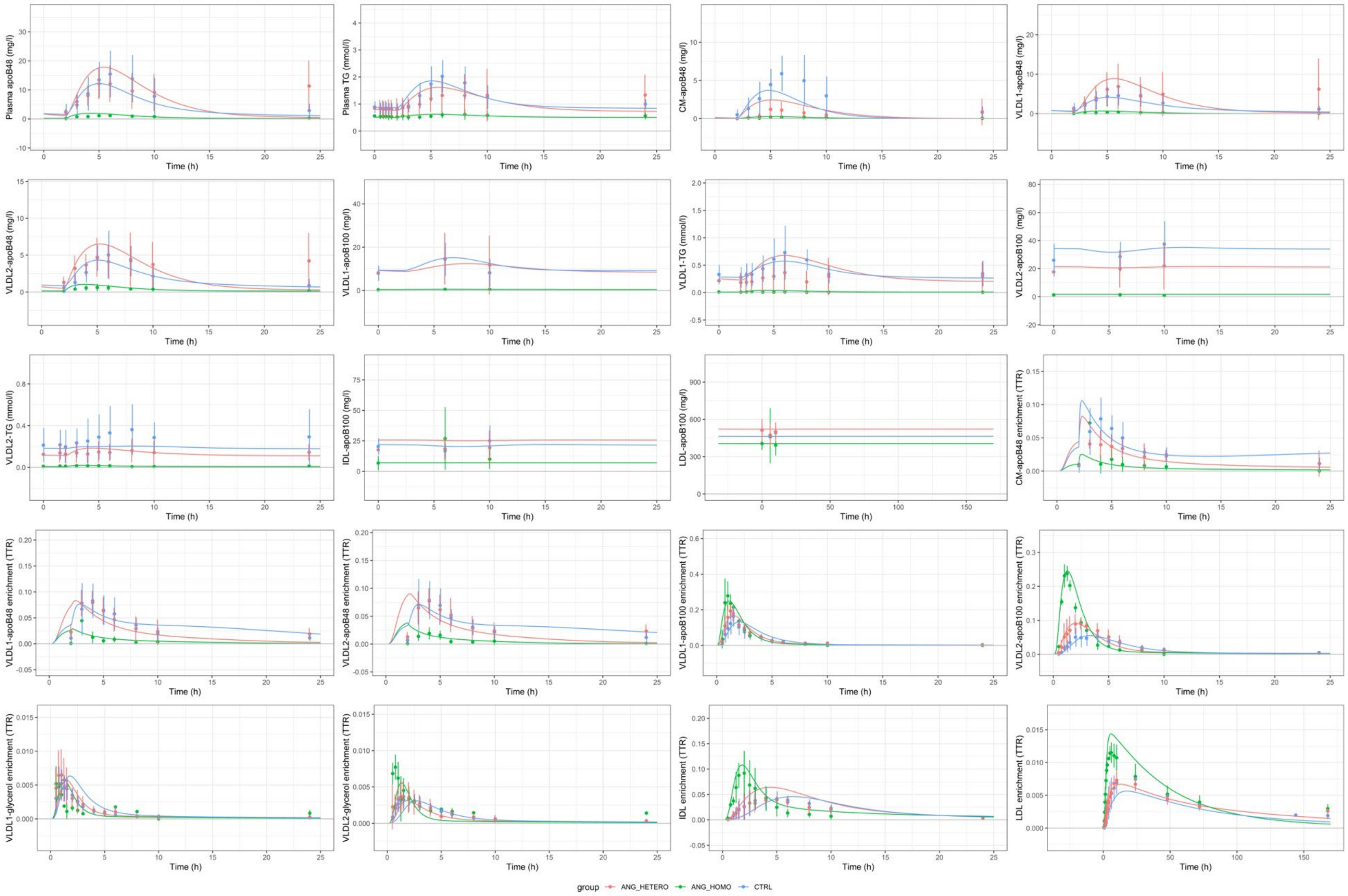

Supplement: Supplementary file 1 — Table S1: Composition of apoB‐containing lipoproteins (mg/mg) in fasting state. Table S2: ApoCIII and apoE fractional clearance rates in ANGPTL3‐deficient and control subjects Figure S1: ApoB concentrations and tracer enrichment in ANGPTL3‐deficient (homozygotes and heterozygotes) and control subjects. [file JOIM-300-312-s001.pdf]
